# Using Stakeholder Engagement to Build Cancer Survivorship Care Coordination Capacity Between Primary and Oncology Care

**DOI:** 10.1177/24731242251385402

**Published:** 2025-11-10

**Authors:** Julie S. Armin, Sarah N. Price, Elizabeth S. Ver Hoeve, Yvonne Bueno, Leila Ali-Akbarian, Nancy Johnson, Elizabeth Calhoun, Lorena Verdugo, Heidi A. Hamann

**Affiliations:** ^1^ Department of Family & Community Medicine, The University of Arizona College of Medicine, Tucson, Arizona, USA.; ^2^ Department of Social Sciences and Health Policy, Wake Forest University School of Medicine, Winston-Salem, North Carolina, USA.; ^3^ Departments of Psychiatry and Family Medicine and Community Health, University of Wisconsin-Madison School of Medicine and Public Health, Madison, Wisconsin, USA.; ^4^ LeCroy & Milligan Associates, Inc., Tucson, Arizona, USA.; ^5^ University of Arizona Health Sciences, The University of Arizona, Tucson, Arizona, USA.; ^6^ University of Illinois Chicago, Chicago, Illinois, USA.; ^7^ El Rio Community Health Center, Tucson, Arizona, USA.; ^8^ Department of Psychology, The University of Arizona, Tucson, Arizona, USA.

**Keywords:** cancer disparities, community-based research, cancer navigation, underserved patients

## Abstract

**Introduction:**

Cancer survivors experience gaps in access to timely and well-coordinated care. We describe stakeholder engagement activities across a university-based comprehensive cancer center and a primary care-focused federally qualified health center, which informed implementation and sustainability of community-focused cancer care coordination interventions to optimize care for underserved cancer patients in specialty and primary care.

**Methods:**

Methods were systematic, iterative, and flexible to establish and maintain trust, honesty, and collaborative decision-making between academic, community, and clinical partners. First, we collaboratively identified clinical (*N* = 43) and patient (*N* = 16) stakeholders and their needs through in-depth interviews and focus groups. Then, we planned, implemented, evaluated, and modified strategies to improve cancer care coordination in response to challenges and new information.

**Results:**

Primary care providers reported needing more information about cancer survivorship care and wanting more complete information about their patients’ cancer treatment. Patients described concerns about cancer recurrence, challenges managing comorbid conditions, and economic concerns. These findings influenced the focus and implementation of a community navigation program at the academic cancer center and informed bi-directional information-sharing interventions with primary and oncology care partners, which included face-to-face meetings, service-learning opportunities, and improving data transmission through the electronic health record.

**Health Equity Implications:**

Health systems’ mutual learning and resource sharing enhance patient cancer care coordination to address social determinants of health. Engaging partners at different levels within health systems (e.g., administrative staff and clinical leadership) ensures sustainability of relationships, interventions, and advocacy for cancer patients with significant social needs.

## Introduction

There are an estimated 18.1 million cancer survivors (defined by the National Cancer Institute as anyone living with a diagnosis of cancer, regardless of treatment status) living in the United States today.^1,2^ Cancer survivors, especially those from historically marginalized groups, experience gaps in well-coordinated care during and after active cancer treatment.^3^ While cancer care coordination interventions show promise for improving patient outcomes,^4^ there are challenges to implementation and sustainability, particularly in different clinical settings (e.g., primary and specialty care) and serving patients with significant social needs.^5–7^

Despite the importance of tailoring cancer care coordination interventions to the clinical setting and patient needs, there is limited research offering a roadmap for establishing effective partnerships and engaging partners in collaborative decision-making about survivorship care coordination. To address this gap, we describe our process of using stakeholder engagement (i.e., cultivating clinical-academic partnerships) to improve care coordination between primary care and cancer care, a connection that could benefit survivorship care.^8^

Involvement of community stakeholders is considered a best-practice framework to guide researchers in developing and implementing culturally responsive interventions.^9^ A “stakeholder” is commonly defined as a representative individual or group directly engaged in or impacted by the action or activity being researched, while “engagement” refers to the bi-directional process through which researchers and stakeholders work together to establish priorities and make decisions about the design, conduct, and use of research.^10^ Soliciting input from stakeholders is associated with increased acceptability, feasibility, and relevance of novel research interventions,^9–11^ thus facilitating the adoption of research into practice^10^ and promoting patient-centered care.^12^

This report outlines our engagement work to inform others who want to establish effective clinical-academic partnerships. As part of a systematic effort to identify stakeholder needs and perspectives and facilitate culturally responsive care coordination interventions, university-based researchers engaged with clinicians and staff at an affiliated^13^ National Cancer Institute-designated comprehensive cancer center (“cancer center”) as well as with staff, administrators, and patients at a federally qualified health center (FQHC) in Tucson, Arizona. The project reported here (2017–2022) employed an action research approach^14^ that includes clinical and patient stakeholder engagement for ensuring an intervention and process that is feasible and sustainable by being responsive to the needs and input of cancer patients, health care providers, clinic staff, and community partners.^15^ Here, we focus on outcomes from our stakeholder engagement process, namely: (1) collaborative identification of stakeholders and needs; and (2) planning, implementing, and evaluating strategies to improve cancer care coordination.

## Methods

Our methods of partner and stakeholder engagement were both systematic and iterative to encourage trust, transparency/honesty, collaborative decision-making, and other best practices of stakeholder engagement.^16^ Quarterly partnership meetings between administrative and clinical leadership at the FQHC and cancer center as well as the university research team continued throughout the duration of the project to maintain transparency.

### Collaborative identification of stakeholders and needs

Initial contact between university researchers, the university-affiliated cancer center, and the FQHC began when investigators approached the FQHC CEO and her team prior to submission of a project proposal to engage the team in proposal development. At project inception, the team formulated a plan to conduct meetings with primary and oncology care administrators and providers at the FQHC and cancer center to plan a community navigation program, which was identified at the time of the proposal submission as a care coordination intervention that we would tailor to the cancer center and FQHC clinical contexts. Stakeholder identification at the cancer center and the FQHC evolved during conversations prior to the submission of the project proposal and continued throughout the project, as the team added new members based on objectives and needs.

Once funding was received, the university team conducted focus groups and in-depth interviews with FQHC and cancer center patients and providers. The formative needs-assessment data collection protocol (IRB# 1702178642, PI: Armin) was reviewed and approved by the University of Arizona’s Institutional Review Board as well as the FQHC’s El Rio Health Patient Safety and Therapeutics Committee, whose charter includes review of all research proposals. The FQHC received a subaward to support the project activities, including the salaries of members of the project team and the cost of supplies for stakeholder engagement activities (e.g., focus groups with health care providers). Patients received $40 in gratitude for their participation in interviews.

At the FQHC, the university team conducted a needs assessment with semi-structured data collection in focus groups (*N* = 32) and in-depth interviews (*N* = 11) with health care providers. Qualitative data were collected regarding providers’ preferences for communication with specialty providers and their approaches to supporting cancer survivors (patients in active treatment and posttreatment). They were also asked to share perspectives on underserved patients’ barriers to cancer survivorship care, since the FQHC is a large safety net provider for the region.

At both the FQHC and the cancer center, English- and Spanish-speaking patients (*N* = 16) were recruited for in-depth interviews to discuss their experiences with cancer, especially with coordinating between primary care and oncology. Patient interviews used purposive sampling to gain understanding of issues faced by diverse patients, especially low-income and/or linguistically minoritized patients.

Audio recordings from these discussions were transcribed and loaded into MAXQDA, a qualitative data management and analysis program.^17^ A code tree was first developed using *a priori* codes (from the literature); the team then identified additional codes from the data after reading through transcripts. Ecosocial theory,^18,19^ which considers historical and social influences on health and lived experience guided coding while allowing for inductive codes (ideas) to emerge from the text. A pragmatic ethnographic approach acknowledged team members’ immersion in their community contexts and facilitated ongoing analysis of interviews during data collection, guiding recruitment to highlight varied experiences.^20^ Two coders applied the codes to the transcripts, and their application was checked for alignment. Any coding discrepancies were discussed until definitions had been refined and coders were able to apply codes consistently.

### Planning, implementing, and evaluating strategies to improve cancer care coordination

The project included regular team meetings, as well as special lectures and cancer-related presentations, to maximize opportunities for building relationships and information sharing between the cancer center and the FQHC. Team meeting frequency and attendance differed based on the clinical site: the university and FQHC team met quarterly; members of the university joined monthly community health worker (CHW) meetings; university team members held frequent, *ad hoc* meetings with cancer center clinical staff, particularly before and during implementation of a patient navigator; and, after implementation, two university team members (EVH and HH) met biweekly with the Supportive Care Team. The Supportive Care Team included social work, nurse navigators, and psychology and psychiatry team members. Documentation of project activities and outcomes provided continuity and data points for evaluation. FQHC and cancer center clinical and administrative stakeholders provided input on research activities, offered insight into the allocation of project resources, and provided a vision for project sustainability.

FQHC stakeholders worked with the research team to establish a timeline, gather feedback from other clinical stakeholders regarding implementation, and develop work plans for the needs assessment and ongoing evaluations. Process data were collected in the form of meeting agendas, notes, and action items and regularly reviewed, discussed, and analyzed by the research team and FQHC stakeholders. One of the team’s interventions, a community navigation program and evaluation, was reviewed and approved by the University of Arizona Institutional Review Board (IRB# 1804483104A005; PIs: Ali-Akbarian & Calhoun).

Below we report the results of stakeholder engagement activities related to identifying clinical needs and implementing the program’s two major interventions to address cancer care coordination. An evaluation of the community navigation intervention has been previously published.^21,22^ Our results section also describes the adjustment of stakeholder engagement activities during the COVID-19 pandemic.

## Results

### Collaborative identification of stakeholders and needs

In focus groups and in-depth interviews, safety-net clinical staff at the FQHC identified barriers to PCP-oncology team-based care and pointed to areas where they needed support in encouraging patient adherence to recommended therapies and survivorship care. Table 1 describes the clinical and/or administrative roles of staff. Both clinical and administrative staff identified general barriers to care experienced by their patients, including language other than English, health literacy, and transportation. FQHC clinical staff made suggestions for expanding survivorship care plans (SCPs) in particular areas by including detail about patient support networks who provide extra social or emotional support to address transportation, health literacy, or other barriers to care. They commented on optimal methods for ensuring delivery of the SCP to the primary care team, including delivering the SCP via the electronic medical record, as to reduce patient burden to facilitate communication.

**Table 1.** Federally Qualified Health Center Data Collection

Staff roles (focus groups)	*N* = 32
Behavioral Health Care Providers	2
Care partners (Community Health Advisors)	3
Health center managers	2
Licensed practical nurses	2
Medical assistants	6
Medical students	3
Nurse managers	1
Physicians (including residents)	7
RN care coordinators	5
Radiology technicians	1

Staff roles (in-depth interviews)	*N* = 11
Behavioral health	1
Clinic manager/Assistant manager	6
Clinical pharmacy	1
Dental	1
Nutritionist	1
Referral clerk	1

English- and Spanish-speaking cancer survivors identified several social and health-related areas of concern pertaining to survivorship, including their changed identity/life after cancer, fear of recurrence, living with co-morbidities (e.g., substance use disorder) and disability, and economic concerns (e.g., job loss, cost of treatment, lack of health insurance coverage). Table 2 outlines participant demographics. Most participants were over the age of 55, and half were enrolled in Medicare. Nearly one-third (31.2%) had either Medicaid or no insurance, and 18.7% conducted their interview in Spanish. Findings from the focus groups, in-depth interviews, and process evaluation (e.g., meeting notes) are included in Table 3. Two areas of interrelated pragmatic intervention were identified through data collection from patients and providers about needs and assets: (a) care coordination to address social determinants of health through bilingual/bicultural community navigation and (b) bidirectional information/data sharing.

**Table 2.** Demographics of Patient Interviewees

Demographic	Characteristics	Frequency (*N* = 16)	Percent (%)
Age	20–45	2	12.5
	45–55	3	18.7
	56–65	4	25.0
	66–^∗^	6	37.5
		1	6.2
Self-reported gender	Female	9	56.2
	Male	6	37.5
	Unknown	1	6.2
Self-reported race	Latino (includes Hispanic, Mexican-American)	5	31.2
	Multiple races	1	6.2
	White (includes Caucasian, European)	9	56.2
	Unknown	1	6.2
Insurance type	Medicaid	4	25
	Medicare	8	50
	None	1	6.2
	Private Insurance	2	12.5
	Unknown	1	6.2
Primary Language of Interview	English	13	81.2
	Spanish	3	18.7

^∗^ 66 years and older.

**Table 3.** Stakeholder Needs and Assets for Coordinating the Care of Underserved Patients

	FQHC	Cancer center	Improvements to care Coordination-Community integration
Cancer Information Needs	Information regarding surveillance and long-term effects of cancer treatment Recommendations for follow-up visits with primary care.		Cancer center bilingual and bicultural community-focused patient navigation Address social determinants of health (SDOH) Connect patients to state health insurance marketplace
Cancer Information Assets	Understand and measure social determinants of health for every patient	Provide resources for cancer community (e.g., support groups)	

	FQHC	Cancer Center	Improvements to Care Coordination- Communication
Patient/ONC/PCP Communication Needs	Lack of communication between ONC and PC — access to electronic data from cancer center — need for cancer center contacts to facilitate timely communication — “black hole” where patient does not return to PCP after oncology care — concern about making patient responsible for sharing information with PCP	Specific clinical contacts at FQHC	Integrate two-way information sharing — providers — navigation/care coordination — connect providers at FQHC and cancer center Improve electronic data sharing between FQHC and cancer center
Patient/ONC/PCP Communication Assets	Use EHR tool for care coordination		

	FQHC	Cancer Center	Improvements to Care Coordination- Patient Centered Care
Patient Centered Care Needs	Patient Needs — Financial assistance — Transportation — Behavioral health — Linguistic needs (Spanish) — Low-literacy — Culturally congruent health/lifestyle recommendations	Lack of access for uninsured patients Need for support of Medicaid patients Lack of Spanish-speaking staff to address patient barriers	Connect patients to PCP for survivorship care Connect to Community Health Advisors at FQHC Expand cancer care to underserved patients Integrate community-focused navigators into clinical team
Patient Centered Care Assets	Electronic tools for care coordination Wrap-around services (e.g., behavioral health, care coordination in PC) Community wellness programming	Creation of survivorship care plans	

ONC, oncology; PCP/PC, primary care provider/primary care.

### Planning, implementing, and evaluating strategies to improve cancer care coordination

While the academic and community partnership had applied for and received a grant to implement a community navigation program at the cancer center, ongoing engagement with community and clinical partners guided program focus and implementation. For instance, a member of the FQHC team (L.V.) helped identify and select community navigators. Further, this engagement helped define the focus of the information-sharing interventions.

#### (a) Care coordination to address social determinants of health through bilingual/bicultural community navigation

The overarching goal of the cancer center’s community-focused patient navigation program was to improve care coordination for underserved patients across the cancer care continuum. Informed by stakeholder feedback and a cancer center needs assessment, the community-focused patient navigation program was designed to facilitate clinical referrals for patients with significant social needs at the time of diagnosis and the transition to survivorship. In response to the needs assessment findings highlighting the needs of Spanish-speaking patients (Table 3), community navigators were selected for their bilingual and bicultural backgrounds, as well as their prior knowledge of community resources. Their 2-day navigator training included 10 modules using adult learning strategies. Additionally, the navigators participated in ongoing training during the study period to meet the needs of each navigator and coordination needs identified through initial qualitative data collection at the FQHC. Activities included compiling a list of community resources to address social needs, attending community events, and regularly meeting with the CHWs at the FQHC and the Supportive Care Team at the cancer center.

At the time of implementation of the cancer center’s navigation program, the navigation co-leads (L.A. and E.C.) held key clinical and research leadership roles at the cancer center. In discussions that commenced months prior to the initiation of community-focused cancer navigation, the team worked through program administration (e.g., space, gaining electronic health record [EHR] access), research needs (e.g., participant recruitment, data collection, and retrieval), community-focused navigator scope of work, and program integration with clinical processes. Cancer center clinical administrators provided input into the navigators’ scope of work and methods for hiring and training the community-focused navigators.

Through conversations with cancer center staff, it was determined that any clinical staff might refer patients to the community-focused navigator program, but that oncology nurse navigators and social workers were most likely to identify patient-reported barriers to care. To remain integrated with the clinical care team, the community-focused navigators attended biweekly supportive care meetings with social work and nurse navigators, along with other clinical staff. Regular meetings with oncology providers and staff at the cancer center occurred throughout project development and implementation and included discussion about resource needs, recruitment flow, the research protocol, and programmatic enhancements. Importantly, *ad hoc* and informal communication with cancer center and FQHC staff, made possible through structured meetings between the research team and stakeholders, enabled program implementation and sustainability.

Any patient who had established care at the cancer center and who had a cancer diagnosis was eligible for participation in the community-focused patient navigation intervention. Once a patient was enrolled in the intervention, the navigators systematically assessed a patient’s psychosocial barriers to the cancer care, worked to address each barrier in a variety of ways, and documented the outcomes of attempts to resolve barriers. Patients participated in the program for approximately 3 months. Results of the intervention evaluation have been published elsewhere, including patient demographics such as cancer stage and treatment phase.^21,22^

#### (b) Bidirectional information/data sharing

Through research team engagement with clinical partners, a variety of methods for bidirectional communication and data sharing were implemented at all levels of health system administration and service provision. One of the FQHC Community Health Workers (CHW, LV) on the study team helped to foster communication between the cancer center’s community-focused navigation program and the FQHC CHWs. The FQHC’s CHWs invited cancer center community-focused navigators to take part in monthly meetings to share community resources, discuss challenges, and facilitate direct referrals between the FQHC and cancer center and vice versa. Furthermore, ongoing discussions between researchers (H.A.H. and E.C.) and partners (N.J. and cancer center administration) about the community-focused cancer navigation program enabled higher level connections for information sharing and patient advocacy between the cancer center and FQHC.

One project discussed by clinical and administrative leadership included the integration of enhanced patient-centered communication via an EHR tool that managed patient referrals from the FQHC to the cancer center, to “close the loop” on timely patient scheduling, a known challenge in health care delivery systems. Furthermore, the cancer center used the shared EHR tool only for receiving referrals, not for communication or for sending referrals, which posed a challenge when the cancer center’s community navigator needed to communicate securely with the FQHC. This project required the team to invite additional stakeholders from information technology at the cancer center and the FQHC to the workgroup. The workgroup connected the director of care coordination at the FQHC with a contact in the clinical network to develop a workflow for bidirectional communication via the shared EHR tool.

In addition to the EHR tool for improving coordination, engagement produced multiple methods of bi-directional communication. At the time of this project, co-author N.J. was CEO of the FQHC; in that role, she prioritized quality, effective cancer survivorship care for the primary care patients from her FQHC. The research team (with N.J as champion) facilitated direct connections between the cancer center and the FQHC’s clinical staff (e.g., mobile numbers). Engagement with clinical staff also included bi-directional service-learning opportunities. Cancer center experts provided training in cancer survivorship care for primary care staff at the FQHC, and FQHC experts offered training for cancer center staff on care coordination using a social determinants of health model.

### COVID-19 Adaptations

The onset of COVID-19 in March 2020 affected information-sharing opportunities, in-person research activities, as well as quarterly partner meeting scheduling. Our medical and clinical partners were called to prioritize COVID-19 clinical and public health responsibilities, and many staff were reassigned to serve the broader community. The cancer center and the FQHC responded to increased clinical responsibilities, and research committee approvals were delayed, which affected the momentum of collaboration activities, including the navigation intervention. Most information-sharing opportunities (e.g., in-service learning), which involved site visits and in-person formats, were postponed, while others resumed as virtual meetings after clinical and administrative staff addressed emergent needs. Community navigation activities continued using virtual meeting tools.

## Discussion

Ongoing partner engagement was essential during a 5-year project to improve cancer care coordination for historically marginalized patients in Southern Arizona, especially in the context of social and clinical contexts that shift frequently. The engagement that built trust, respect, and transparency among partners was especially important during the COVID-19 public health emergency, which caused the research team to pivot its approaches to our two focus areas: care coordination to address social determinants of health and bidirectional information/data sharing.

Patient navigation programs in oncology care were developed to improve patient access to oncology services, particularly for underserved patients^23,24^ and through the use of community navigators, nurses, or teams of health care professionals.^25^ The community-focused patient navigation program described here was implemented as a component of care coordination, a collaborative approach to assess and meet patients’ clinical and community needs at the point of diagnosis and into survivorship.^26^ While the research team systematically collected data to inform the navigation program and other interventions, informal, *ad hoc* and iterative conversations were also essential in successful and sustainable implementation. For instance, our navigation team co-lead (L.A.) was able to have informal discussions with key stakeholders (e.g., lead nurse navigator) about the community-focused navigation project during routine, and often informal, discussions about patient care in the cancer center. These conversations enabled the team to identify ideal methods of the community-focused navigators’ interactions with nurse navigators and social workers,^21^ including recruitment/referral, consent, and documentation. Hence, a patient navigation intervention, which was planned for implementation prior to receiving program funding, was tailored to the local clinical and community context through ongoing stakeholder engagement.

Furthermore, mutual learning and resource sharing opportunities provided an opportunity to enhance patient cancer care coordination and address social determinants of health. Results of this work suggest that proactive processes for improved communication between oncology providers and community health centers are needed to provide comprehensive, multidisciplinary care and optimal care coordination for underserved patients. Bringing in stakeholders as needed (e.g., IT staff) and facilitating communication and problem-solving may address intractable issues, such as data sharing between different institutional EHRs. The shared cancer survivor population may be facing unique challenges that make them particularly socially and structurally vulnerable due to economic disadvantages, discrimination, comorbidities, and multiple barriers in access to care. Engagements and resource sharing between specialty and primary care may offer innovations in health care quality through enhanced referrals or other improvements in care coordination, including the transition back to primary care from oncology care.^27–29^

Finally, engaging many partners in health care systems—from high-level administrators to clinical staff—may ensure that connections between institutions can be maintained despite projects ending or staff attrition. This engagement of partners in many roles can also help address program sustainability through creative problem-solving related to infrastructure and funding.^13^ These partnerships led to additional funding to address cancer care for uninsured patients at a community-wide level and a permanent position for a community navigator in the cancer center clinic.^21^ The navigation program outcomes provided compelling data for the health system to invest in supporting a navigator for their underserved patients.

## Limitations

Although the university team assessed access to cancer care through an evaluation of patient outcomes by demographic categories in the cancer center medical record (e.g., adherence to treatment appointments by insurance type), there was a missed opportunity to systematically collect input from cancer center clinical staff about patient needs. Instead, we used an *ad hoc* approach—catching them in meetings to ask questions—and relied on our team member (“key informant,” L.A.), who was also a clinician at the center. This oversight is instructive, as it highlights how academia often conceptualizes community as “other,” rather than attending to communities within. The university runs the research-focused cancer center, while the clinical enterprise is owned and operated by a private nonprofit entity in partnership with the university.^13^ Upon reflection, we concede there would have been project benefits through systematic data collection from clinical stakeholders at the cancer center, which might have given clinicians space to identify areas of concern about patient care that did not come up in project-focused discussions.

## Abbreviations Used

CEO: Chief executive officer
CHW: Community health worker
FQHC: Federally qualified health center
ONC: Oncology
PCP: Primary care provider
PC: Primary care
